# Apelin regulates FoxO3 translocation to mediate cardioprotective responses to myocardial injury and obesity

**DOI:** 10.1038/srep16104

**Published:** 2015-11-06

**Authors:** Frederic Boal, Jessica Roumegoux, Chiara Alfarano, Andrei Timotin, Denis Calise, Rodica Anesia, Anne Drougard, Claude Knauf, Christine Lagente, Jerome Roncalli, Franck Desmoulin, Helene Tronchere, Philippe Valet, Angelo Parini, Oksana Kunduzova

**Affiliations:** 1 National Institute of Health and Medical Research (INSERM) U1048, Toulouse, Cedex 4, France; 2 University of Toulouse, UPS, Institute of Metabolic and Cardiovascular Diseases, Toulouse, France; 3 US006, Microsurgery Services, Toulouse, Cedex 4, France; 4 Department of cardiology, Toulouse University Hospital, Toulouse, Cedex 9, France

## Abstract

The increasing incidence of obesity accentuates the importance of identifying mechanisms and optimal therapeutic strategies for patients with heart failure (HF) in relation to obesity status. Here, we investigated the association between plasma level of apelin, an adipocyte-derived factor, and clinicopathological features of obese and non-obese patients with HF. We further explored potential regulatory mechanisms of cardiac cell fate responses in conditions combining myocardial injury and obesity. In a prospective, cross-sectional study involving patients with HF we show that obese patients (BMI ≥30 kg/m^2^) have higher left ventricular ejection fraction (LVEF) and greater levels of plasma apelin (p < 0.005) than non-obese patients (< 30 kg/m^2^), independently of ischemic etiology. In a mouse model combining ischemia-reperfusion (I/R) injury and high-fat diet (HFD)**-**induced obesity, we identify apelin as a novel regulator of FoxO3 trafficking in cardiomyocytes. Confocal microscopy analysis of cardiac cells revealed that apelin prevents nuclear translocation of FoxO3 in response to oxygen deprivation through a PI3K pathway. These findings uncover apelin as a novel regulator of FoxO3 nucleocytoplasmic trafficking in cardiac cells in response to stress and provide insight into its potential clinical relevance in obese patients with HF.

Cardiovascular disease and obesity are common, interrelated health problems in industrialized societies. In the general population, obesity is considered to be a risk factor in the development of heart failure and is traditionally regarded to impact negatively on the outcome of myocardial ischemia. However, despite the evidence for a higher prevalence of obesity in myocardial ischemia patients, a number of recent publications suggested that obesity in humans with ischemic heart disease is associated with reduced morbidity and mortality, the so-called “obesity paradox”^1^,^2^,^3^. The mechanism underlying this paradox is complex and remains unclear.

Many lines of evidence suggest that mitochondrial dysfunction, including mitochondrial loss and the production of reactive oxygen species (ROS) may be important in the development and progression of heart failure^4^,^5^. Excessive generation of ROS in the heart can directly lead to increased necrosis and apoptosis of cardiomyocytes which subsequently induce cardiac dysfunction^6^,^7^. The mitochondria are both the primary sources of ROS and the primary targets of ROS damage under pathological conditions including myocardial ischemia/reperfusion (I/R)^8^. The central role of mitochondria-derived ROS in the obesity-related processes and cardiac I/R-induced damage has been shown recently^9^,^10^. However, the mechanisms through which mitochondrial ROS may regulate cell fate decisions in response to stress remain poorly defined.

The forkhead box O (FoxO) family of transcription factors plays a fundamental role in the regulation of mitochondrial activity and cellular responses to oxidative stress. In mammals, the FoxO family comprises four isoforms (FoxO1, FoxO3, FoxO4, and FoxO6) characterized by an overlapping expression during development and in adulthood. FoxO acts as transcriptional activators governing a variety of vital cellular processes including cell survival, apoptosis, metabolism, DNA repair and resistance to oxidative stress. A number of studies point toward a role for FoxO3 in maintaining cardiovascular and metabolic homeostasis^11^,^12^. Although the existing data are controversial, there is some evidence that FoxO3 regulates life/death decisions in response to cellular stressors. In cardiac microvascular endothelial cells FoxO3 leads to ROS accumulation, and in parallel, induces the disturbance of Bcl-2 family proteins which results in activation of apoptosis^13^. In contrast, in cardiomyocytes, knockdown of endogenous FoxO3 sensitizes cells to undergo apoptosis, whereas enforced expression of FoxO3 inhibits apoptosis^14^. Additional work in animal models of age-dependent oxidative stress responses demonstrated that up-regulation of FoxO3 in the heart and adipose tissue is associated with activation of cellular antioxidant systems^15^. Trafficking of FoxO between the nucleus and the cytoplasm plays a decisive role in stress-related regulation of cell death/cell survival. Shuttling in and out of the nucleus not only provides a mechanism to control signal-dependent access of proteins to nuclear targets, but also contributes to regulate the activity of proteins in the cytoplasm^16^. Given the fundamental role of FoxO3 in cardiovascular and metabolic homeostasis, the function and fine-tuned regulation of its subcellular distribution is critical for the future development of specific and effective cardioprotective therapeutics in clinical situations combining obesity and myocardial damage.

The adipose tissue acts as an endocrine organ, and plays a substantial role in the pathogenesis of cardiovascular and metabolic diseases^17^. Altered levels of adipocyte-derived factors or “adipokines”, may be particularly related with heart disease^17^,^18^. We have previously demonstrated that apelin, a recently described adipokine, plays an important role in the regulation of cardiovascular and metabolic homeostasis^19^,^20^. Apelin, an endogenous ligand for the G-protein-coupled receptor APJ, exerts strong inotropic activity and increases coronary blood flow by vascular dilation^21^. In response to pathological stress, apelin-APJ axis regulates myocardial remodeling and cardiac function^20^,^22^. The loss of apelin impairs I/R remodeling and exacerbates myocardial I/R injury *ex vivo* and *in vivo*^23^. Apelin-deficient mice develop age-related progressive cardiac dysfunction which is prevented by apelin infusion^24^, suggesting an important role of apelinergic system in maintaining cardiac performance. In humans, circulating and cardiac levels of apelin are reduced in patients with acute myocardial infarction and established coronary artery disease^25^,^26^,^27^. In explanted human hearts, the primary ischemic injury is associated with the loss of apelin in various compartments confirming an important role of apelin in human heart failure^23^. By contrast, in obese subjects, plasma apelin concentrations are increased^28^,^29^. However, the role of apelin in conditions combining myocardial infarction and obesity remains to be determined.

## Materials and Methods

### Reagents and antibodies

Antibodies used in this study are: anti-FoxO3 (75D8) from Cell Signalling Laboratories, anti-tubulin from SantaCruz Biotechnologies; anti-beta-actin from Sigma (A1978). Fluorescent Alexa-coupled secondary antibodies were from Life Technologies and HRP-coupled secondary antibodies from Cell Signaling Technologies. DAPI was from Life Technologies. Apelin-13 was purchased from Bachem and was referred to as apelin throughout this study. All other chemicals were from Sigma unless otherwise stated. siRNA against FoxO3 were from Eurogentec and were as follows: 5′-GGAUAAGGGCGACAGCAAC-3′ and 5′-GUUGCUGUCGCCCUUAUCC-3′. The dominant-negative mutant FoxO3-TM plasmid was kindly provided by Dr A. Dejean (Physiopathology Center of Toulouse-Purpan, Toulouse, France) and was as described^30^.

### Primary mice cardiomyocytes preparation, cell culture, transfection and treatments

Adult mice ventricular cardiomyocytes were isolated from adult obese mice and maintained as described^31^. The rat embryonic cardiomyoblastic cell line H9C2 was cultured in MEM medium (Gibco 41090–028) supplemented with 10% FBS and 1% penicillin-streptomycin in a 37 °C, 5% CO_2_ incubator. siRNA transfection was performed with Lipofectamine RNAiMAX (Life Technologies) according to manufacturer’s instructions. H9C2 cells were transfected using JetPrime (Polyplus Transfection) according to manufacturer’s instructions. For transfection studies, FoxO3-TM was co-transfected with peGFP-C1 at a ratio of 80:20 to identify transfected cells. Cells were pretreated for 15 minutes with apelin (10^−7^ M) and then subjected to normoxia (5% CO_2_; 21% O_2_, balance N_2_) or hypoxia for 2 hours in a hypoxic chamber (5% CO_2_, 1% O_2_, balance N_2_). The dose of apelin was chosen on the basis of our previous *in vitro* studies^20^,^22^. To measure cell apoptosis induced by hypoxia, the cells were left for 16 hours in hypoxic conditions.

### Evaluation of apoptosis

Apoptosis level both *in vivo* and *in vitro* was assessed using the DeadEnd Fluorometric TUNEL system according to manufacturer’s instructions (Promega) as described before^20^.

### Hydrogen peroxyde and superoxide production

Mitochondrial O_2_^-^ and H_2_O_2_ production in cells was measured by MitoSOX (Life Technologies) and MitoPY1 (Sigma-Aldrich) at 1 μM (on H9C2 cells) or 5 μM (on cardiomyocytes) for 30 min following live-cell imaging on a confocal microscope equipped with an incubation chamber with temperature control and CO_2_ enrichment. H_2_O_2_ level in hearts were measured by amperometry as described^32^ and LPO (lipid hydroperoxyde) quantification was done as described before^22^.

### Caspase-3, plasmatic troponin I and metabolic measurements

Caspase-3 activity was assessed with EnzChek Caspase-3 Assay Kit #1 (Life Technologies) according to the manufacturer’s instructions. Plasmatic Troponin-I (Life Diagnostics) were quantified using specific ELISA kits according to the manufacturers’ instructions. Insulinemia (Mercodia) and glycemia (Accu-check, Roche Diagnostics) were measured in fasted state. Body fat mass composition was determined as described before^31^.

### Animal studies

The investigation conforms to the Guide for the Care and Use of Laboratory Animals published by the US National Institutes of Health (NIH Publication No. 85–23, revised 1985) and was performed in accordance with the recommendations of the French Accreditation of the Laboratory Animal Care (approved by the local Centre National de la Recherche Scientifique ethics committee). Wild**-**type male C57BL6/J mice purchased from Janvier Labs or apelin-KO mice (generated as described^31^) were fed a high fat diet (HFD, 45% fat) for 20 weeks, corresponding to the acquisition of an obese and insulin resistant phenotype^31^,^32^. The metabolic profile of HFD-fed mice is summarized in Table 1. Apelin KO mice were as described before^31^.

### Experimental protocol

A mouse model of ischemia-reperfusion (I/R) was used as previously described^33^. In brief, the mice were intubated and placed under mechanical ventilation after undergoing general anesthesia with an intraperitoneal injection of ketamine HCl (35 mg/kg) and xylazine (5 mg/kg). A left parasternotomy was performed to expose hearts, and a 7–0 silk suture (Softsilk) was placed around the left anterior descending coronary artery. A snare was placed on the suture, and regional myocardial ischemia was produced by tightening the snare. After 45 minutes of ischemia, the occlusive snare was released to initiate reperfusion up to 24 hours. Sham-operated control mice underwent the same surgical procedures except that the snare was not tightened. Animals were randomly divided into four groups: (I) sham vehicle (*n* = 6), (II) ischemia-reperfusion (I/R) vehicle (*n* = 7), (III) sham apelin (*n* = 7), and (IV) ischemia-reperfusion apelin (*n* = 7). Mice received intravenously apelin (0.1 μg/kg) or vehicle (PBS) at 5 min of reperfusion in a final volume of 100 μl. The dose of apelin was selected on the basis of our preliminary animal studies.

### Determination of area at risk and infarct size

Determination of area at risk and infarct size was done as described before^33^. Briefly, at the end of the infarction protocol, in some animals of each group, the left coronary artery was reoccluded, and 1 mL of 1.5% Evans blue dye was injected into the left ventricular cavity to measure the myocardial ischemic area at risk. The animals were euthanized immediately, and the heart was removed and cut from apex to base in 4 to 5 transverse sections. After incubation in 1% triphenyltetrazolium chloride (TTC) solution in isotonic pH 7.4 phosphate buffer at 37 °C for 20 minutes, the slices were subsequently fixed in 10% formalin solution for 6 hours to assess myocardial tissue viability and determine myocardial infarct size. Evans blue stained area represents non-ischemic tissues, TCC stained zone (red) indicates ischemic but viable tissues while white unstained area represents the necrotic/ischemic tissues. Infarct size was expressed as a percentage of the ischemic risk area.

### Immunofluorescence

Immunofluorescence was performed essentially as previously described^34^. Briefly, cells grown on glass coverslips were PFA-fixed and permeabilized using TritonX-100 before incubation with primary and secondary antibodies, mounted in Mowiol and imaged using confocal microscopy on a Zeiss LSM780 microscope. For nuclear FoxO3 quantification, the fluorescence intensity of FoxO3 proteins in the nucleus was quantified and normalized against the fluorescence intensity within the total cell.

### Immunolabeling of heart sections

Paraformaldehyde**-**fixed (4%) and paraffin embedded heart sections were deparaffinized and rehydrated, antigen retrieval was performed using a sodium citrate treatment. Alternatively, serial cryosections (10 μm) immobilized on Superfrost Plus slides (Thermoscientific) were rehydrated in PBS, fixed in 4% PFA for 10 min. Permeabilization of cardiac tissues was performed with 0.2% Triton X-100 for 20 min. After blocking of non specific sites with 1% BSA, the primary antibodies were incubated o/n at 4 °C. After labeling with appropriate secondary antibodies, the sections were mounted in Vectashield mounting medium including DAPI (Vector Laboratories) and imaged by confocal microscopy.

### Morphology

Ultrastructural studies of cardiac tissues by electron microscopy were done as before^31^. Briefly, cardiac tissues were fixed in cold 2.5% glutaraldehyde/1% paraformaldehyde, post-fixed in 2% osmium tetroxide, embedded in resin, and sectioned. Cardiac mitochondrial number relative to the section area was determined from electron micrographs as described previously^35^.

### Real-time RT–PCR analysis

Total RNAs were isolated from cultured mouse cardiac fibroblasts using the RNeasy mini kit (Qiagen). Total RNAs (300 ng) were reverse transcribed using Superscript II reverse transcriptase (Invitrogen) in the presence of a random hexamers. Real-time quantitative PCR was performed as previously described^31^. The expression of target mRNA was normalized to GAPDH mRNA expression. The sequences of the primers used are as follow and given in the 5′-3′ orientation:

FoxO3, sense GCAAAGCAGACCCTCAAACTG, antisense TGAGAGCAGATTTGGCAAAGG; GAPDH, sense TGCACCACCAACTGCTTAGC, antisense GGCATGGACTGTGGTCATGAG; Bax, sense CGGCGAATTGGAGATGAACT, antisense GTCCACGTCAGCAATCATCCT; Bcl-2, sense TCCCGATTCATTGCAAGTTGTA, antisense GCAACCACACCATCGATCTTC; ANP, sense AGAGTGGGCAGAGACAGCAAA, antisense AAGGCCAAGACGAGGAAGAAG; IL-6, sense GCCCACCAAGAACGATAGTCA, antisense CAAGAAGGCAACTGGATGGAA. The content of mitochondrial DNA (mtDNA) was calculated using real-time quantitative PCR by measuring the threshold cycle ratio of a mitochondrial encoded gene (COX1) and a nuclear-encoded gene (cyclophilin A) as previously described^35^.

### Population and plasma samples collection

Forty patients, aged 43 to 82 years, with heart failure (HF) were included in this observational study. Patients were recruited from the cardiology department at the Toulouse-Rangueil University hospital, France from May 2013 to January 2014. CHF patients had known stable HF with more than 3 months without any decompensation episodes, irrespective of clinical severity (stage II to IV of NYHA classification) and etiology. Diagnosis of heart failure had been formally established by a cardiologist from clinical observations, heart disease follow-up, transthoracic echocardiography (TTE) for all patients and BNP monitoring. These patients were included during their regular scheduled visit at the hospital. The study was approved by a local ethics committee and included only patients who provided written informed consent. The research protocol conforms to the ethical guidelines of the 1975 Declaration of Helsinki. Patients were stratified according to their obesity status (obesity defined by a BMI ≥ 30 kg/m^2^). Thus, 13 were enrolled in the obese group and 27 were enrolled in the non-obese group. Peripheral venous blood from the cohort subjects was collected into EDTA tubes. After centrifugation at 1500 g at 4 °C for 10 min, plasma was separated and stored at −80 °C until assayed.

### Statistical analysis

Data are expressed as mean ± SEM. Comparison between two groups was performed by Student’s t-test while comparison of multiple groups was performed by one-way ANOVA followed by a Bonferroni’s post hoc test using GraphPad Prism version 5.00 (GraphPad Software, Inc). Statistical significance was defined as p < 0.05 unless otherwise stated in figure legends.

For clinical studies, data were presented as mean values ± SD or when the data failed the D’Agostino-Pearson test for normal distribution as median with 95% confident interval (CI) for continuous variables and as percentage for categorical variables. For categorical variables, a Pearson Chi-square test was used to determine the statistical significance of the association between the variable and obesity status. For continuous variables, a Student’s *t-*test or Mann-Whitney rank sum test when normality test failed was used to determine the statistical significance of the association between the variable and obesity status with a 2-tailed P value determination. Plasma apeline concentrations were analyzed by two-way ANOVA followed by Bonferroni’s post-hoc analysis using GraphPad Prism (version 5.0) software (GraphPad Software, San Diego, CA, USA). P < 0.05 was considered statistically significant. Logistic regression analysis was performed using Statistical R (version 3.0.1; http://www.r-project.org, MASS package version 7.3.37) to analyze the relationship between obesity or ischemic heart failure status and apelin. Two methods were used to select independent variables in the model: enter method, all variables were included without checking; stepwise method, variables were sequentially included, check and possibly remove variables that became non-significant after entering a variable (enter if P < 0.1 remove if P > 0.2).

## Results

### Plasma apelin levels and cardiac functions in obese and non-obese patients with HF

**Figure 1:**
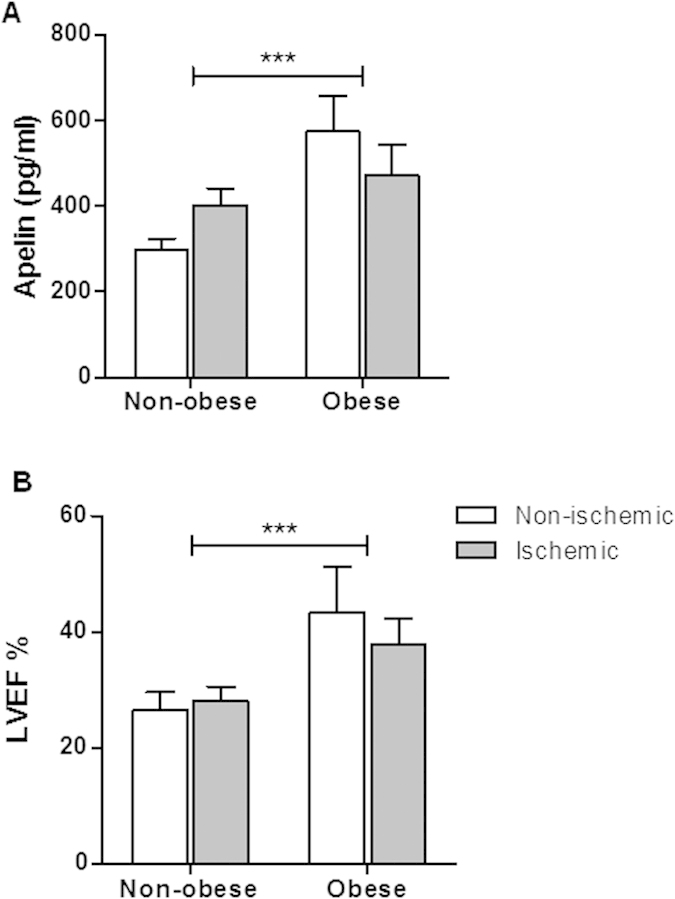
Apelin plasmatic levels and LVEF in patients with HF. (**A**) Plasma apelin concentrations in obese and non-obese patients. Data are presented as mean ± SEM. ***p < 0.005, two way Anova/Bonferroni’s post-test for pair-wise comparison of obese and non-obese patients. p = 0.946, two way Anova/Bonferroni’s post-test for pair-wise comparison of non-ischemic vs ischemic cardiomyopathy. (**B**) Left ventricular ejection fraction (LVEF) values in obese and non-obese patients. Data are presented as mean ± SEM. ***p < 0.005, two way Anova/Bonferroni’s post-test for pair-wise comparison of obese and non-obese patients. p = 0.562, two way Anova /Bonferroni’s post-test for pair-wise comparison of non-ischemic vs ischemic HF.

Apelin plasma levels are increased in obese individuals^28^,^29^, whereas circulating apelin levels are reduced in patients with HF^25^,^26^,^27^. In order to evaluate whether plasma levels of apelin differ among patients with HF in relation to obesity status, patients were divided into 2 categories based on BMI: non-obese and obese patients. Demographic and clinical data of these patients are shown in Table 2. The ischemic cardiomyopathy constituted 60% of all causes of HF. The mean apelin concentration was 401 ± 172 pg/ml in the cohort. Plasma levels of apelin were significantly higher in obese than in non-obese HF patients (503 ± 202 and 352 ± 134 pg/ml, p < 0.005, respectively). Plasma apelin level measured in obese patients was independent of the ischemic status (Fig. 1A). Indeed, the multivariate analysis of the association of apelin including sex, age, LVEF, T2-diabetes and ischemic HF with obesity gave odds ratio (OR) of 3.95 (95% CI, 1.31-11.94), p = 0.015 and 2.82 (95% CI, 1.23–6.47) per 100 pg/ml, p = 014 by using enter and stepwise computing method, respectively whereas association of apelin with ischemic heart failure status remained not significant, p = 0.566 (Table 3). Mean LVEF value was significantly higher in obese patients (Fig. 1B). The higher mean LVEF value measured in obese patients was independent of the ischemic heart disease status (Table 3).

### Effect of apelin on cardiac apoptosis, infarct size and inflammation in a mouse model combining I/R injury and obesity

**Figure 2:**
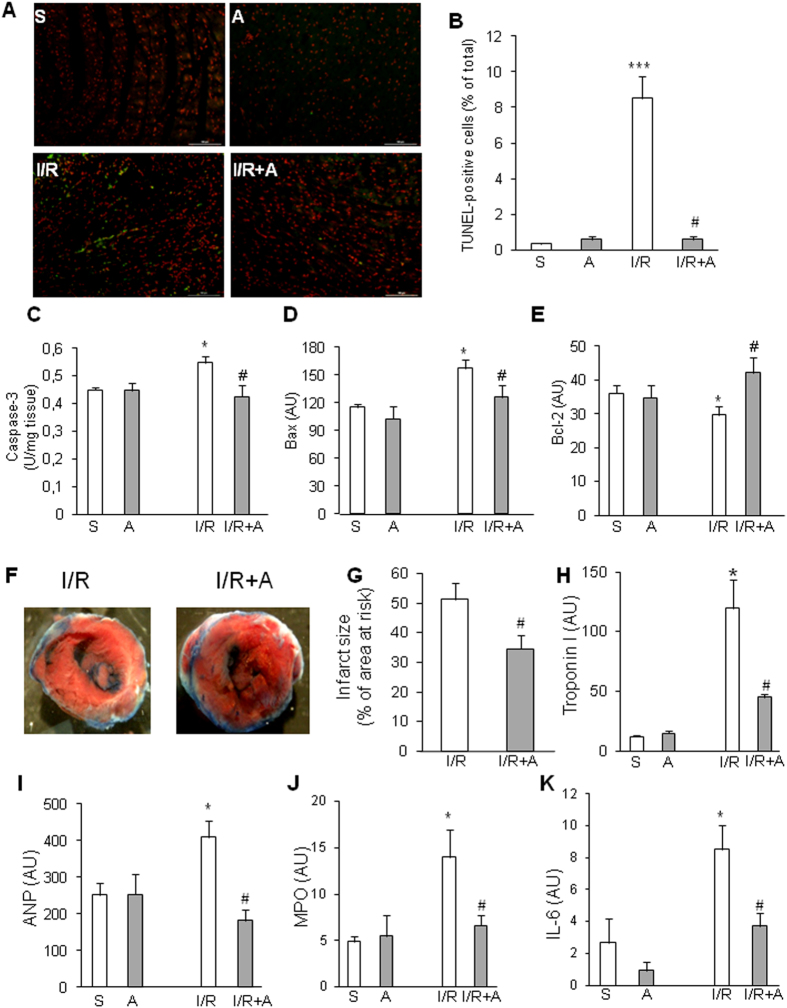
Apelin reduces cell death, infarct size and inflammation in obese mice hearts after I/R surgery. (**A**) TUNEL staining of heart sections from obese mice treated or not with apelin (**A**) and sham-operated (S) or subjected to I/R. Apoptotic cells are labeled in green, nuclei are stained in red. (**B**) Quantification of apoptosis from (**A**). The percentage of apoptotic cells was quantified for each condition. (**C–E**) Caspase-3 activity (**C**), Bax (**D**) and Blc-2 (**E**) expression levels in hearts from obese mice treated as indicated. *p < 0.05 compared with S; ^#^p < 0.05 as compared with I/R. (**F**) Cross-sections of hearts treated as indicated were stained with Evans blue and TTC. (**G**) Quantification of infarct size expressed as percentage of area at risk. ^#^p < 0.05 as compared with I/R. (**H–K**) Troponin I plasmatic levels (**H**), ANP expression level (**I**), MPO activity (**J**) and IL-6 expression level (**K**) in hearts of obese mice treated as indicated. ***p < 0.001 vs S; *p < 0.05 vs S; ^#^p < 0.05 vs I/R.

Myocardial infarction is the most frequent cause of heart failure^36^. We next examined physiopathological features and effects of post-treatment with apelin in a mouse model combining I/R injury and HFD-induced obesity. In cardiac tissue from HFD-fed mice subjected to 24h I/R, TUNEL assay revealed a significant increase in apoptotic cells as compared to sham-operated animals (Fig. 2A,B). Myocardial apoptosis was further confirmed by analysis of caspase-3 activation and apoptosis-related proteins. Apelin post-reperfusion treatment of I/R mice reduced caspase-3 activity (Fig. 2C) and expression of Bax (Fig. 2D) as compared to vehicle-treated mice after I/R. Conversely, Bcl-2 expression level, an anti-apoptotic protein, was increased in apelin-treated I/R hearts (Fig. 2E). As compared to vehicle-treated HFD-fed I/R mice, the infarct area was significantly reduced in apelin-treated animals compared to vehicle I/R animals as shown visually and quantitatively in Fig. 2F,G, respectively. Apelin-dependent cardiac protection was accompanied by a reduction in troponin I, a specific indicator of cardiac damage (Fig. 2H), and in ANP level (Fig. 2I). We also evaluated the cardiac expression of inflammatory factors in mice subjected to sham or I/R. Apelin-treated mice had significantly decreased levels of MPO activity (Fig. 2J) and IL-6 level (Fig. 2K) compared with vehicle-treated I/R mice.

### Apelin post-treatment prevents mitochondrial damage after I/R injury in HFD-induced obesity

**Figure 3:**
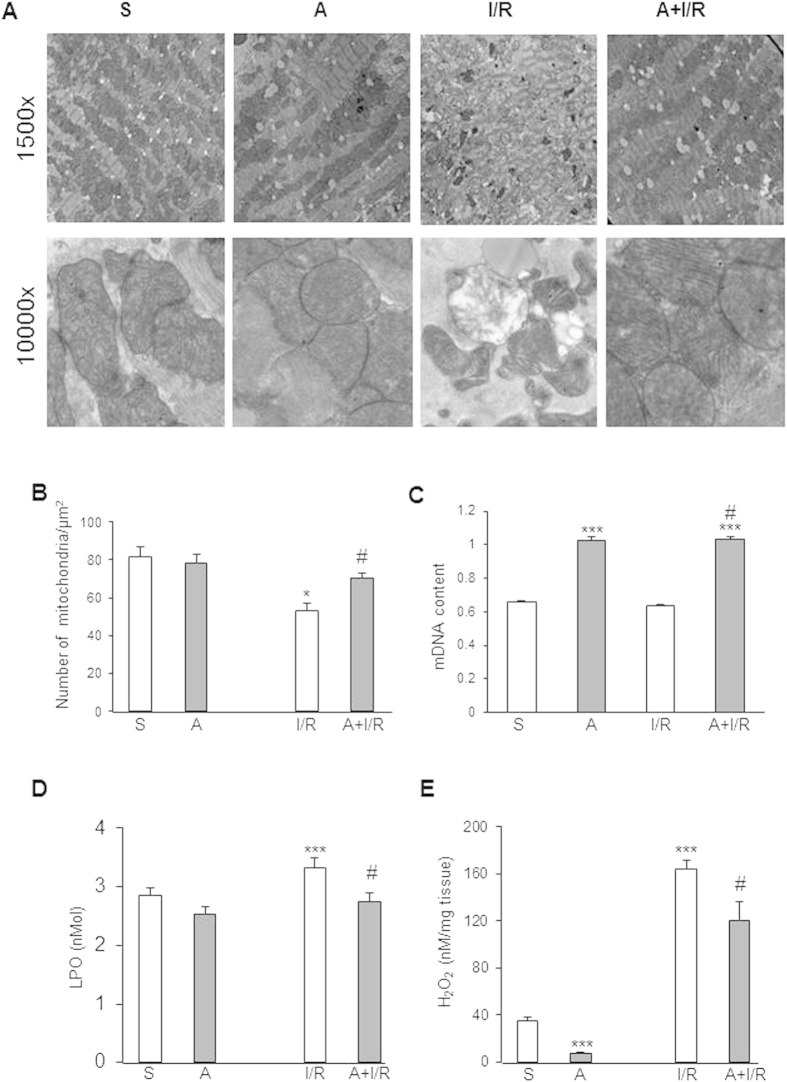
Apelin-treatment protects from mitochondrial damage, H2O2 production and lipid peroxidation in obese heart. (**A**) Typical electron micrographs from sham (S), apelin-treated (**A**) or animals subjected to I/R are shown at original magnifications x1500 and x10000. (**B**) Quantitative analysis of mitochondrial density in heart tissues. (**C**) qRT-PCR analysis of mtDNA content. (**D,E**), Plasmatic LPO (**D**) and H_2_O_2_ levels in cardiac tissues (**E**) from obese mice treated as indicated. *p < 0.05; ***p < 0.001 vs S; ^#^p < 0.05 vs I/R.

Given the central role for mitochondria in ROS production and cell fate decisions, we next examined mitochondrial ultrastructure, density and DNA content in HFD-fed mice after 24h I/R. Electron microscopy analysis revealed I/R-induced mitochondrial damage including swelling and structural alterations, as compared with sham-operated mice (Fig. 3A). As shown in Fig. 3B, tissue sections from HFD-fed hearts subjected to I/R exhibited a 39% decrease in mitochondrial density as compared to control sham mice. There were no differences in mtDNA content between HFD-fed sham and I/R mice (Fig. 3C). Importantly, plasma concentration of LPO, a marker of oxidative stress (Fig. 3D), and cardiac H_2_O_2_ level (Fig. 3E) were markedly increased after I/R in HFD-fed mice as compared to control group. Remarkably, apelin post-reperfusion treatment significantly prevented mitochondrial ultrastructural damage (Fig. 3A,B), increased mtDNA content (Fig. 3C) and reduced myocardial LPO (Fig. 3D) and H_2_O_2_ (Fig. 3E) levels in HFD conditions after I/R.

### Apelin reduces hypoxia-induced mitochondrial ROS and apoptosis through the FoxO3 pathways.

**Figure 4:**
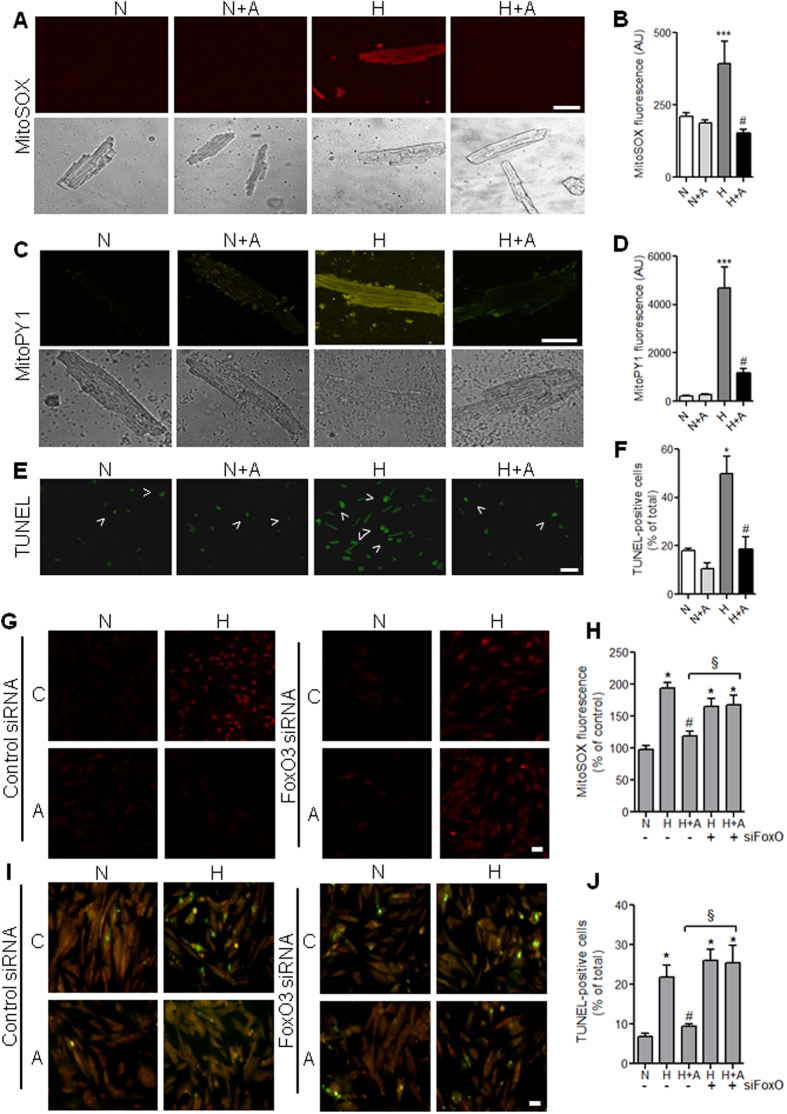
Apelin treatment reduces hypoxia-induced ROS production and apoptosis in cardiomyocytes through the FoxO3 pathway. Primary cardiomyocytes isolated from HFD-fed mice were treated with apelin for 15 minutes and submitted to hypoxia for 2 h. (**A**–**F**) Mitochondrial O_2_^−^ (**A,B**) and H_2_O_2_ (**C,D**) production were followed using the fluorescent probes MitoSOX Red and MitoPY1 respectively. Apoptosis was measured by TUNEL labeling (**E,F**). Arrowheads highlight TUNEL-positive cells. Bar is 20μm in all panels. (**G,H**) H9C2 cardiomyoblasts were transfected with siRNA targeting FoxO3 or with scramble control siRNA, treated or not (**C**) with apelin (A, 10^−7^M for 15 minutes) and subjected to hypoxia (**H**) or normoxia (**N**) for 2 hours. Mitochondrial ROS production was measured by MitoSOX Red fluorescence (in red). (**I,J**) H9C2 cells were treated as in (**G**) and apoptotic cells were labeled with TUNEL staining. Bar is 20μm. *p < 0.05 vs N; ***p<0.001 vs N; ^#^p < 0.001 vs H; ^§^p < 0.05 between indicated conditions.

In order to evaluate mitochondrial ROS production in conditions combining cardiomyocytes damage and obesity, we measured mitochondria-specific superoxide (O_2_^-^) and H_2_O_2_ generation by MitoSOX and MitoPY1 probes, respectively, in cardiomyocytes isolated from HFD-fed mice under hypoxia. Analysis of mitochondria-specific ROS formation after 2h of hypoxic stress demonstrated that apelin treatment attenuated hypoxia-induced mitochondria-specific O_2_^-^ (Fig. 4A,B) and H_2_O_2_ generation (Fig. 4C,D). Moreover, in cardiomyocytes derived from HFD-fed mice, hypoxia-induced apoptosis was significantly reduced by apelin treatment (Fig. 4E,F).

FoxO3 is known to be critical for the regulation of oxidative stress and apoptosis in cells^13^. Therefore, we investigated if apelin’s ability to reduce cell death and oxidative stress in cardiac cells was FoxO3-dependent. Silencing of FoxO3 by siRNA abolished the ability of apelin to attenuate hypoxia-induced mitochondrial O_2_^-^ production in cardiomyoblasts (Fig. 4G,H). In addition, FoxO3 knockdown drastically blocked apelin-mediated anti-apoptotic activity in response to hypoxia (Fig. 4I,J).

**Figure 5:**
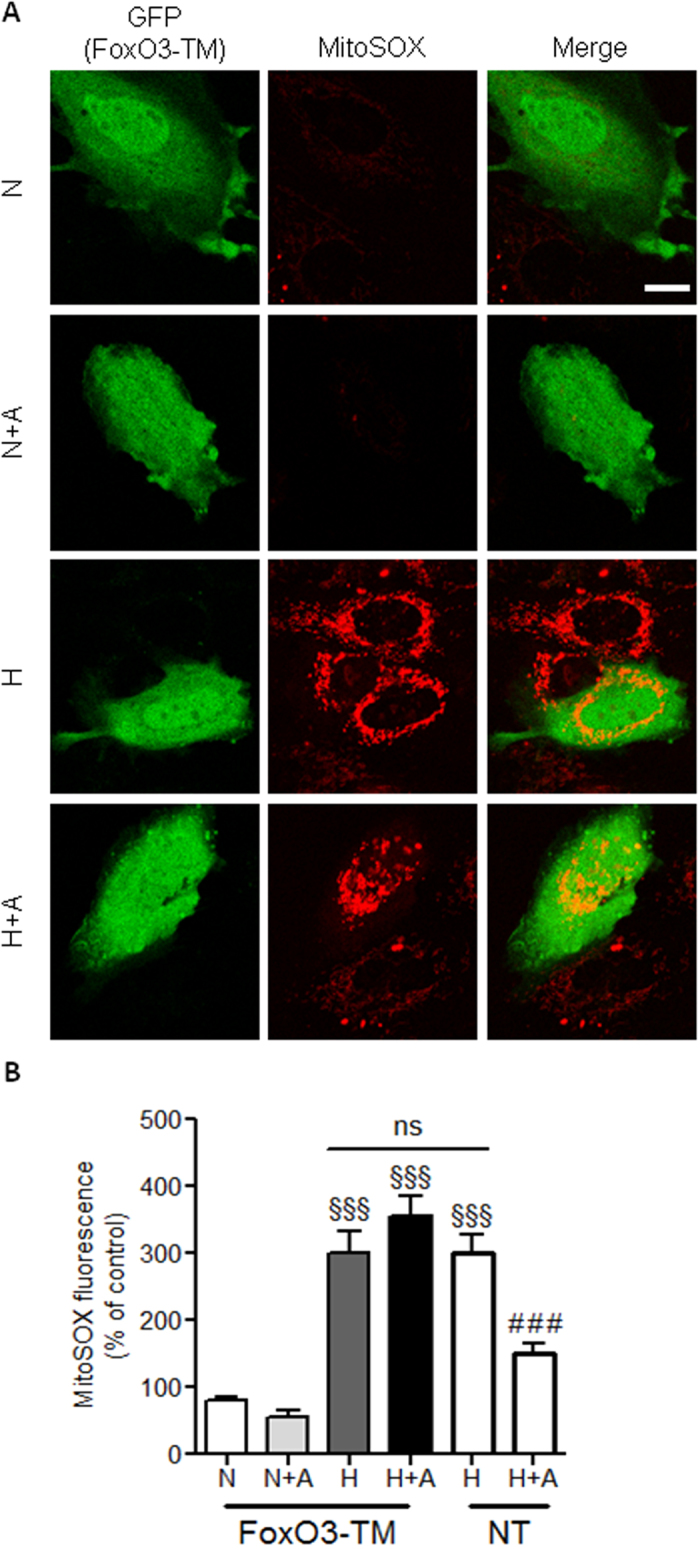
Abbrogation of anti-oxidant effect of apelin by overexpression of FoxO3-TM in H9C2 cells. (**A**) H9C2 cells were transfected to express GFP (in green) and the dominant negative mutant FoxO3-TM. Cells were submitted to hypoxic conditions (**H**) or kept in normoxia (**N**) in the presence or not of apelin (**A**). Mitochondrial O^2−^ production was measured by confocal microscopy using MitoSOX (in red) in transfected cells and in non-transfected cells (NT) as a control. Bar is 10μm. (**B**) Quantification of MitSOX fluorescence per cells from (**A**). ^§§§^p < 0.001 vs N from transfected cells; ^###^p < 0.001 vs H from NT cells; ns, non significant.

In order to confirm the implication of FoxO3 in apelin’s ability to reduce mitochondrial ROS production, we overexpressed the constitutively active FoxO3-TM mutant. As shown in Fig. 5A,B overexpression of FoxO3-TM in H9C2 cells did not result in significant increase in mitochondrial O_2_^-^ as compared with non-transfected cells (Fig. 5A,B). Strikingly, overexpression of FoxO3-TM abolished the ability of apelin to reduce mitochondrial O_2_^-^ generation in response to hypoxia (Fig. 5A,B).

### FoxO3 nucleocytoplasmic shuttling is regulated by apelin in cardiac cells in response to oxidative stress through a PI3K pathway

**Figure 6:**
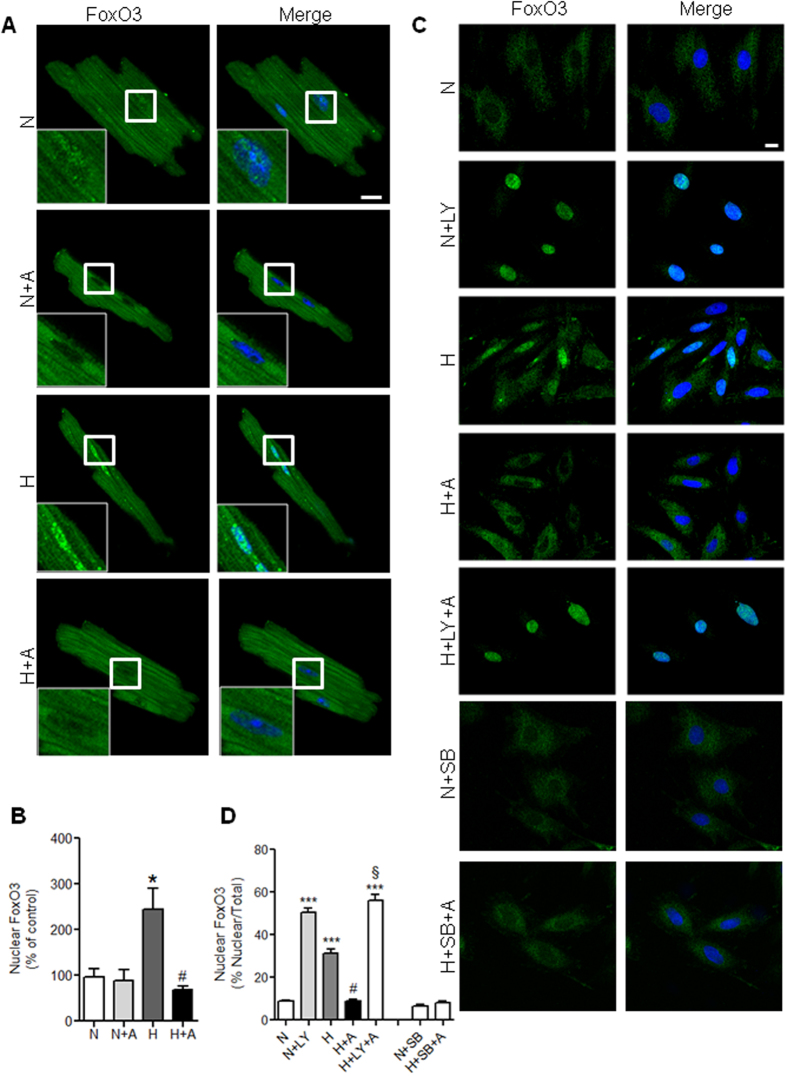
Apelin treatment reverses FoxO3 nuclear translocation induced by hypoxia in cardiac cells through a PI3-K dependent pathway. (**A**) Representative confocal images of cardiomyocytes isolated from HFD-fed mice stimulated or not by apelin for 15 minutes (A, 10^−7^M), submitted to hypoxic (**H**) or normoxic (**N**) conditions for 2 hours, and stained in green for FoxO3. Nuclei were stained with DAPI (in blue). Bar is 20 μm. (**B**) Quantification of FoxO3 nuclear translocation from (**A**,**C**) H9C2 cells treated with the PI3-K inhibitor LY294002 (LY) or with the p38/MAPK SB203580 (SB) in the presence (**A**) or absence of apelin, and then submitted to hypoxic conditions (H) or left in normoxia (**N**) for 2 h. Cells were fixed and stained with an anti-FoxO3 antibody (in green), nuclei were stained with DAPI (in blue). Bar is 10 μm. (**D**) Quantification of FoxO3 nuclear translocation was done as in (**B**). *p < 0.05 vs N; ***p < 0.001 vs N; ^#^p < 0.001 vs H; ^§^p < 0.001 vs H+A.

The subcellular trafficking of FoxO factors is one of the key aspects to control cell fate decisions^37^. We next examined the nucleocytoplasmic dynamics of FoxO3 in cardiomyocytes isolated from HFD-fed mice in response to hypoxia. Positive staining with anti-FoxO3 antibodies was identified in cardiomyocytes isolated from HFD-fed mice (Fig. 6A), where FoxO3 was found in the cytoplasm in normoxia (21% O_2_). Exposure of cardiomyocytes to hypoxia (1% O_2_) for 2 h resulted in predominant nuclear translocation of FoxO3 as demonstrated by the strong colocalization with DAPI (Fig. 6A). Strikingly, apelin treatment completely prevented hypoxia-induced nuclear translocation of FoxO3 (Fig. 6A,B) suggesting that apelin coordinates FoxO3nucleocytoplasmic trafficking in response to stress.

**Figure 7:**
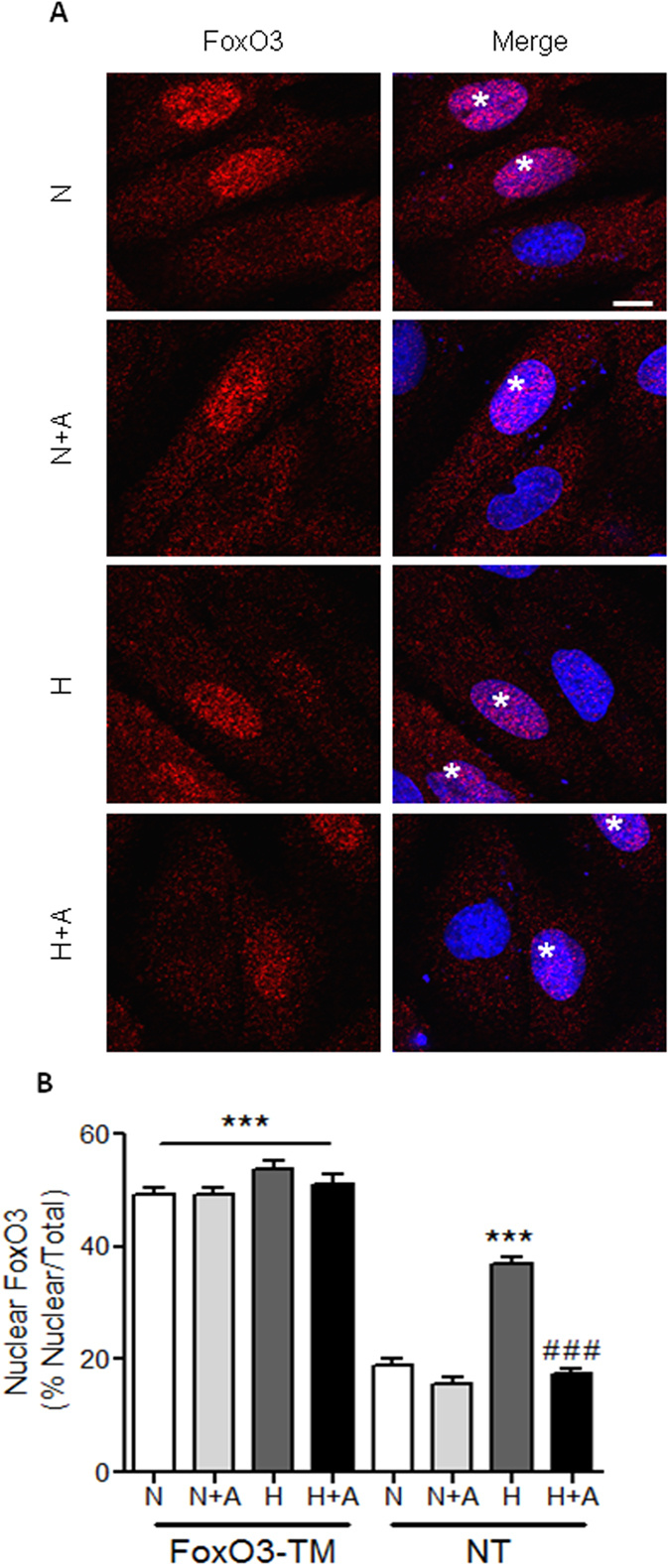
Apelin is not able to induce the nuclear exclusion of FoxO3-TM in H9C2 cells. (A) H9C2 cells overepressing the dominant negative mutant FoxO3-TM were submitted to hypoxic conditions (H) or kept in normoxia (N) in the presence or not of apelin (A), fixed and stained with an anti-FoxO3 antibody (in red). Nuclei were stained with DAPI (in blue). Imaging parameters were set to easily identify cells overexpressing FoxO3-TM (highlighted by stars in merge pictures). Bar is 10μm. (B) Quantification of FoxO3 nuclear accumulation in both untransfected cells (NT) and cells expressing FoxO3-TM. ***p < 0.001 vs N from non-transfected cells (NT, endogenous FoxO3); ^###^p < 0.001 vs H from NT cells.

To elucidate the potential mechanisms of FoxO3 trafficking in response to oxidative stress, we examined whether PI3K/Akt or p38/MAPK are involved in hypoxia-dependent FoxO3 nuclear translocation. As shown in Fig. 6C in H9C2 cells, we observed the same profile of FoxO3 nuclear translocation as in isolated cardiomyocytes. Inhibition of the PI3K pathway by LY294002 in normoxic H9C2 cells resulted in a selective FoxO3 nuclear accumulation (Fig. 6C and quantified in Fig. 6D). Strikingly, LY294002 treatment completely abolished apelin effect on hypoxia-induced FoxO3 nuclear localization (Fig. 6C,D). However, inhibition of the p38/MAPK pathway by SB203580 did not abrogate the effect of apelin (Fig. 6C,D). In order to confirm the implication of PI3K/Akt pathway in apelin-dependent FoxO3 nuclear translocation, we resorted to the use of the non-phosphorylatable FoxO3-TM mutant. As shown in Fig. 7, this mutant is constitutively localized in the nuclei in H9C2 cells in basal condition. Strikingly, apelin was not able to induce the nuclear exclusion of FoxO3-TM both in normoxia and hypoxia (Fig. 7A,B), confirming that apelin prevents hypoxia-induced FoxO3 nuclear translocation through a PI3K/Akt dependent pathway.

### Attenuation of FoxO3 nuclear translocation by apelin in response to I/R injury in obese conditions in mice.

**Figure 8:**
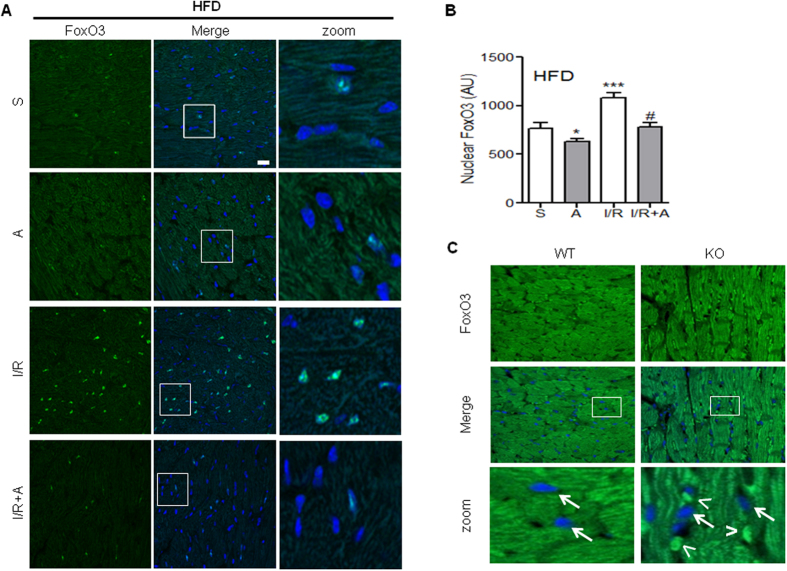
FoxO3 nuclear translocation in mice hearts subjected to I/R is abrogated by post-reperfusion injection of apelin. (**A**) Heart sections from HFD-fed mice treated or not with apelin (**A**) and sham-operated (S) or submitted to I/R were immunostained for FoxO3 (in green). Nuclei were stained with DAPI (in blue). Bar is 20 μm. (**B**) Quantification of FoxO3 nuclear translocation from (**A**). *p<0.05 vs S ***p < 0.001 vs S; ^#^p < 0.05 vs I/R. (**C**) Immunostaining of FoxO3 (in green) on heart sections from HFD-fed WT or apelin KO mice. Nuclei were stained with DAPI (in blue). Arrows point to nuclei devoid of FoxO3, whereas arrowheads point to nuclear staining.

Consistent with the *in vitro* results, analysis by confocal microscopy of cardiac tissue from HFD-fed mice subjected to I/R showed an increased nuclear staining for FoxO3 compared to sham-operated mice (Fig. 8A and quantified in Fig. 8B). Strikingly, apelin post-reperfusion treatment prevented FoxO3 nuclear translocation after I/R injury (Fig. 8A,B). To confirm the role of apelin in cardiac FoxO3 regulation, we examined its subcellular localization in left ventricles from apelin KO HFD-fed mice. As shown in Fig. 8C, apelin deficient mice displayed a FoxO3 nuclear targeting phenotype, as compared to WT mice.

## Discussion

In the present work, we provide the first line of evidence that in obese patients with HF greater apelin levels are associated with the higher LVEF independently of ischemic etiology. In addition, using *in vitro* and *in vivo* approaches, we identify apelin as a novel regulator of FoxO3 nucleocytosolic trafficking in cardiomyocytes in conditions combining myocardial injury and obesity. In many cell types, FoxO3 translocates to the nucleus in response to stress where it activates genes directly involved in cell-fate decisions such as apoptosis, survival and oxidative stress status^38^. Our results suggest that prevention of I/R-induced FoxO3 nuclear translocation by apelin is associated with activation of survival pathways and cardioprotection in obese mice after ischemic injury.

FoxO3 transcription factor orchestrates a number of vital processes involving cell survival, apoptosis, metabolism and stress responses^39^,^40^. The evidences that subcellular localization of FoxO transcription factors is critical for fundamental cellular functions have been extensively reported in non-myocyte cells^16^,^41^. However, in cardiomyocytes FoxO3 trafficking and regulation remain to be elucidated. In the present study we revealed that FoxO3 translocation is regulated by apelin through a PI3K-dependent mechanism in cardiac cells. Indeed, pharmacological inhibition of PI3K and expression of the non-phosphorylatable FoxO3 mutant resulted in the loss of apelin’s ability to control FoxO3 translocation. Analysis of cardiomyocytes isolated from HFD-fed mice demonstrated that hypoxic stress triggers FoxO3 nuclear translocation. These data are consistent with the results reported in recent studies showing that FoxO3 is sensitive to hypoxia, and is mainly localized in the nucleus under conditions of cellular stress in non-myocyte cells^42^,^43^. Consistent with the *in vitro* results, nuclear FoxO3 localization was observed in cardiac tissue from I/R mice under HFD conditions. Most importantly, our *in vitro* and *in vivo* results suggest that apelin promotes the retention of FoxO3 in the cytoplasm by preventing its nuclear translocation in response to stress. Indeed, in cardiac cells and mice exposed to stress apelin was able to inhibit nuclear accumulation of Foxo3. Furthermore, we show that in apelin KO mice cardiac FoxO3 is mainly nuclear, suggesting that apelin plays an important role in FoxO3 nucleocytoplasmic distribution in the heart. Together, these data demonstrate that subcellular localization of FoxO3 is animportant indicator of cell fate decisions in response to stress. Indeed, FoxO3 nuclear accumulation is associated with activation of cell death cascades and ROS overproduction, whereas, cytoplasmic retention of FoxO3 may reflect activation of survival-promoting pathways. In mice, FoxO1/FoxO3 deficiency in cardiomyocytes results in increased myocardial cell death, reduced cardiac performance and increased scar formation following myocardial infarction^12^. Cardiac I/R injury in mice with cardiac FoxO deficiency is accompanied by reduced expression of antioxidants, DNA repair enzymes and antiapoptotic genes^12^. In our study we demonstrated for the first time that inhibition of hypoxia-induced FoxO3 nuclear translocation by apelin is associated with reduced mitochondrial O_2_^-^ and H_2_O_2_ generation *in vitro* and *in vivo* models. Given the importance of FoxO family in the regulation of redox status, FoxO3 subcellular localization may be an important indicator of mitochondrial oxidative stress in cardiac cells. This notion is corroborated by a recent study demonstrating that FoxO3 is an early biomarker of oxidative stress in conditions of metabolic stress^44^. In our *in vitro* experiments we showed that silencing of endogenous FoxO3 or overexpression of FoxO3-TM did not induce ROS production in basal condition. This is in line with a recent report showing that in neonatal cardiomyocytes constitutive activation of FoxO3 did not alter basal level of ROS and apoptosis^12^. Importantly, epigenetic inhibition of FoxO3 or expression of the mutant FoxO3 abolished apelin-dependent cell protection from excessive mitochondrial ROS production, suggesting that the apelinergic system plays an important role in cell resistance to stress.

Mitochondrial ROS are highly reactive and can cause irreversible oxidative damage to the mtDNA, proteins and lipids, but are also involved in signaling from the mitochondria to the cytoplasm. Excessive mitochondrial ROS production triggers activation of cellular apoptotic programs leading to cell death^45^. Limiting mitochondrial ROS production by apelin may be particularly important in cardioprotection against I/R damage. Indeed, we show that apelin-dependent reduction in oxidative stress is associated with reduced infarct size, inhibition of apoptotic cell death and inflammation in HFD-fed mice subjected to I/R. Perhaps the most significant finding from our study is that post-treatment with apelin preserves mitochondrial ultrastructural integrity and increases mtDNA content in conditions combining I/R injury and obesity. Structural defects of mitochondria in cardiac tissue is a major determinant of myocardial injury in chronic heart failure and obesity^4^,^5^,^31^. Since mitochondria are major sources for ROS production and energy generation, any structural and functional alterations of mitochondria may lead to impaired mitochondrial biogenesis and oxidative stress. Recently, we have demonstrated that the perturbations in myocardial energy metabolism play a central role in cardiac performance during the transition from adaptation to maladaptation of the heart in obese state^31^ High-energy requiring cells, such as cardiomyocyte, require large quantities of ATP and maintain high mtDNA content^5^. Our data suggest that regulation of mtDNA content and mitochondrial integrity by apelin may play an important role for maintaining cellular bioenergetics and mitochondrial redox processes in response to myocardial I/R.

Regulation of mitochondrial function is an important role of FoxO factors in coordinating cellular adaptation to hypoxia^46^. Moreover, apelin decreased myocardial expression of pro-apototic protein Bax and increased expression of anti-apoptotic Bcl-2. Interestingly, inhibition of apoptotic signaling was associated with reduced myocardial markers of oxidative stress suggesting that apelin activates survival pathways conditions combining cardiac I/R injury and obesity. In accordance with *in vivo* evidence, we found that higher levels of plasma apelin are associated with a higher LVEF in obese patients with HF as compared to non-obese patients with HF. Our data are in line with previously reported clinical data demonstrating that obese patients with established cardiovascular disease might have better short- and long-term prognosis, suggesting an “obesity paradox”^47^,^48^. In chronic HF, convincing evidence has accumulated from studies including > 30 000 patients over a broad spectrum of disease severity, that being overweight is associated with decreased mortality^49^,^50^. Similarly, in patients with acutely decompensated heart failure, higher BMI is associated with lower in-hospital mortality^51^. Our data suggest that high plasma concentrations of apelin in obese patients with HF are associated with better cardiac performance. However, the sample size of our study is relatively small which may impact on our power to detect more subtle differences between obese and non-obese phenotype. Future studies with larger sample sizes would needed to confirm our findings.

Taken together, these results unravel a novel function of apelin in the regulation of FoxO3 nucleocytoplasmic trafficking and may provide new insights into the mechanistic basis of obesity paradox.

## Additional Information

**How to cite this article**: Boal, F. *et al.* Apelin regulates FoxO3 translocation to mediate cardioprotective responses to myocardial injury and obesity. *Sci. Rep.* **5**, 16104; doi: 10.1038/srep16104 (2015).

## Figures and Tables

**Table 1:** Metabolic parameters of mice under ND or HFD feeding.

	ND	HFD
Body weight (g)	28.5 ± 0.6	47.5 ± 0.4***
Fat mass (%)	14.1 ± 0.9	38.8 ± 1.2***
Glycemia (mM)	8.0 ± 0.2	10.9 ± 0.8***
Insulinemia (pg/ml)	1545.7 ± 293.2	3753.3 ± 591.8**

Male C57BL/6J mice were fed ND or HFD for 20 weeks. Body weight, fat mass, and plasma parameters were measured at the end of week 4. Data are means ± sem; n = 8 per group. **p  < 0.01 and ***p  < 0.001 vs ND-fed group.

**Table 2:** Characteristics of the patients with HF.

	All HF	non-obese HF	obese HF	
	(N = 40)	(N = 27)	(N = 13)	P
Age, y	64 ± 9	63 ± 10	66 ± 7	0.305
Sex, Female, % (F/M)	12 (5/35)	15 (4/23)	8 (1/12)	0.653
BMI	27.6 ± 5.1	24.8 ± 2.6	33.2 ± 4.6.	<**0.001**
Cardiovascular risk factors				
Hypertensive, % (n)	40 (16)	41 (11)	39 (5)	1.000
T2-Diabetes, % (n)	35 (14)	22 (6)	61 (8)	**0.031**
Dyslipedemia, % (n)	45 (18)	37 (10)	61 (8)	0.185
Smoking, % (n)	10 (4)	11 (3)	8 (1)	1.000
Heart failure etiology				
Ischemic CM, % (n)	60 (24)	56 (15)	69 (9)	0.503
Valvular CM, % (n)	5 (2)	7 (2)	0 (0)	0.550
Dilated CM, % (n)	35 (14)	37 (10)	31(4)	0.740
Medication				
ACEIs, % (n)	72 (29)	74 (20)	69 (9)	1.000
ARBs, % (n)	15 (6)	11 (3)	23 (3)	0.370
Beta-blockers, % (n)	90 (36)	92 (25)	85 (11)	0.583
Diuretics, % (n)	67 (27)	74 (20)	53 (7)	0.100
Vitamin K antagonists, % (n)	45 (18)	52 (14)	31 (4)	0.312
Antiplatelet agents, % (n)	67 (27)	63 (17)	77 (10)	0.484
Statines, % (n)	65 (26)	59 (16)	77 (10)	0.316
Admission labs				
BNP, pmol/ml	450 [245–957]	614 [326–1487]	214 [127–774]	0.073
Creatinine, μmol/l	101 [96–124]	103 [93–134]	100 [82–143]	0.817
C reactive protein, mg/l	7.8 [3.5–11.6]	8.1 [2.7–13.6]	7.8 [3.3–14.7]	0.858
Na^+^ mM	138 ± 3	138 ± 3	138 ± 3	0.523
ALT, U/ml	29 [25–36]	26 [19–38]	32 [26–48]	0.311
Admission vitals				
Mean Blood Pressure, mmHg	86 ± 12	83 ± 11	91 ± 12	**0.035**
Heart rate, Bpm	73 ± 16	75 ± 16	70 ± 17	0.449
Echocardiography				
LVEF, %	31 ± 13	27 ± 10	39 ± 13	**0.003**
LVEF < 30%, % (n)	42 (17)	52 (14)	23 (3)	0.103
NYHA class				
II, % (n)	60 (24)	55 (15)	69 (9)	0.503
III, % (n)	35 (14)	37 (10)	31(4)	0.740
IV, % (n)	5 (2)	4 (1)	8 (1)	1.000

CHF, cardiac heart failure; BMI, body mass index; CM, cardiomyopathy; ACEIs, angiotensin-converting enzyme inhibitors; ARBs, angiotensin II receptor blockers; BNP, B-type natriuretic peptide concentration; ALT, alanine aminotransferase; LVEF, left ventricular ejection fraction; NYHA class, New York Heart Association functional classification.

**Table 3:** Multivariate analysis association between plasma apelin level, obesity and ischemic HF.

	OR^a^	95% CI	P	OR^b^	95% CI	P
Association with Obesity						
Apelin per 100 pg/ml	3.95	1.31–11.94	0.015	2.82	1.23–6.47	0.014
Sex, Female = 1	17.20	0.21–1394	0.205	E		
Age per 10 years	1.87	0.54–6.42	0.322	E		
LVEF per 5%	2.46	1.20–5.08	0.014	2.26	1.20–5.08	0.015
Ischemic HF =1	1.71	0.10–29.54	0.710	E		
T2-Diabetes =1	32.00	1.60–635	0.024	13.23	1.38–126	0.025
Association with Ischemic HF^c^						
Apelin per 100 pg/ml	1.15	0.71–1.86	0.566	E		
Sex, Female = 1	0.18	0.20–10.79	0.179	E		
Age per 10 years	1.74	0.79–3.81	0.166	E		
LVEF per 5%	0.95	0.68–1.32	0.766	E		
Obese =1	1.47	0.20–10.79	0.706	E		
T2-Diabetes =1	0.68	0.13–3.46	0.645	E		

OR, odds ratio; CI, confident interval, LVEF, left ventricular ejection fraction.

^a^enter method: all variables where included without checking. P < 0.0005 for the association with obesity; p = 0.3414 for the association with Ischemic HF.

^b^stepwise method: variables were sequentially included in the model, variable with p > 0.2 were excluded (E).

^c^population was dichotomized according the cardiovascular artery disease status (Ischemic HF, n = 24; without ischemic HF, n = 16 patient).
